# Book review of ‘Disaster by choice: How our actions turn natural hazards into catastrophes’

**DOI:** 10.4102/jamba.v12i1.996

**Published:** 2020-09-14

**Authors:** Gideon van Riet

**Affiliations:** 1 School of Government Studies, Faculty of Humanities, North-West University, Potchefstroom, South Africa

This engaging book makes the argument that disasters are not natural and that humans can prevent them, if we choose to do so. The author is well qualified to comment on this topic. Ilan Kelman is a professor of Disasters and Health at the University College London, London, United Kingdom, and a professor at the University of Agder, Kristiansand, Norway.

The book is filled with rich examples and details of specific historical events. The author explains how these have been the result of natural hazards and human vulnerability. In many instances, he explains how vulnerability reduction was possible, but not sufficiently executed. In other cases, he analyses and warns of potential threats currently not adequately addressed and how vulnerability to these hazards might and should be reduced. The central argument of this concise 167-page book appears to be that manipulating natural hazards instead of working with these hazards to reduce human vulnerability increases the risk of complacency and as such major catastrophe. These catastrophes are the result of choices, although not typically the choices of the most vulnerable. Instead, it is the choices made by those in positions of relative power that eventually place the most marginal members of society at risk. It seems this book is especially aimed at a general readership. The central argument is framed as somewhat ‘radical’, although those who have been studying disasters, even only briefly, would know that the argument is instead a concise summary of the dominant paradigm in disaster reduction. Nonetheless, I still believe the book would be useful to more seasoned students of disaster, a point to which I will return. As it appears aimed at a more general readership, the text is sparingly referenced, with around eight pages of notes and another half a page of recommended reading.

The first two chapters are the highlights of the book. These well-polished discussions draw the reader in through case studies such as the earthquake, subsequent cholera outbreak, and humanitarian misdeeds by UN forces in Haiti in 2010. The argument here is that all of this could have been prevented through proper planning and execution. In a similar vein, chapter two deals with the opposites of water and fire as hazards, based on the examples of wildfires in Australia and the United States and the prospect of flooding in Singapore and Canvey Island in England. Kelman demonstrates how natural hazards present communities and authorities with choices. The best choices are to reduce vulnerability to known hazards.

Chapters three and four deal with the concept of vulnerability. The organisation of these two chapters is somewhat unusual. Perhaps by attaching the content to more established categories, and more categories in general, the book could have better clarified what is at times quite an elusive concept. Instead, categories such as vulnerability by ideology, vulnerability by economics and vulnerability by numbers are used. The reading experience is a bit more laboured. However, these chapters still convey the gist of the concept at an appropriate level for novice readers.

Chapter five is entitled ‘making the choice’ and chapter six is entitled ‘making the change’. The former deals with the complexity of certain contexts. Issues such as ‘unknown unknowns’ and complexity, which sometimes make choices difficult, are acknowledged. It is important that a book of this nature includes such qualifications. Still, Kelman argues that disasters are mostly the result of human choices and can therefore be averted and mitigated. Chapter six provides examples of successful risk management. Toronto, as case in point, has been relatively successful. In that city, flooding as a hazard has been acknowledged and vulnerability reduction measures are in place. Even though these are not infallible, they greatly reduce the risk of a major catastrophe. This and other examples substantiate Kelman’s essential argument that people making the right kind of choices reduce disasters.

Aimed at a more general readership, many will likely find the book insightful. As a contribution within disaster studies, it is still a useful book by a global expert, although it might reveal a challenge currently facing the field, which is in no way an indictment of Kelman. Why, seemingly, have we been making the same argument, or a version thereof, for almost 50 years? Is it because the proverbial penny has not yet dropped with enough important decision-makers? This is almost certainly part of the answer. However, disaster scholars may also ask whether it is because they are unsure how to progress from here, based on the state of knowledge? We know the struggle is largely political. Kelman too tells us this much. But, how do at-risk communities and disaster scholars engage power from what is seemingly a relative deficit? Kelman, in a sense, provides a useful account, or bookmark if you like, of the current state of knowledge. Disaster scholars might, however, want to consider whether this succinct argument for vulnerability reduction should not also serve as an important bookend. My point is not that we abandon the vulnerability paradigm. Few serious scholars of disaster would find such a notion reasonable. Instead, we might want to flesh out this perspective even more, both conceptually and in terms of praxes. Investigating the micro-politics of public safety in specific contexts is one possible point of departure. There may of course be many others.

This puzzle aside, Kelman’s succinct and generally lucid account of the state of knowledge within the field, will likely be useful to a wide range of readers. These include the general public. Social scientists, engineers, city planners and natural scientists who would like to understand how their fields relate to the problem of disaster and why this is important would also find the book quite illuminating. Moreover, the generous use of examples will be valuable to students of disaster, also as a teaching aid. Finally, the book would be of significant interest to senior undergraduate and graduate students, academics and practitioners interested in entering the field of disaster studies. It offers an accessible point of entry into the dominant and arguably still most plausible perspective within the field.

